# Kaempferitrin-Treated HepG2 Differentially Expressed Exosomal Markers and Affect Extracellular Vesicle Sizes in the Secretome

**DOI:** 10.3390/biom11020187

**Published:** 2021-01-29

**Authors:** Wei-Chi Ku, Badrinathan Sridharan, Jiann-Yeu Chen, Jen-Ying Li, Shu-Yu Yang, Meng-Jen Lee

**Affiliations:** 1 School of Medicine, College of Medicine, Fu Jen Catholic University, New Taipei 242062, Taiwan; 089052@mail.fju.edu.tw; 2 Department of Applied Chemistry, Chaoyang University of Technology, Taichung 413310, Taiwan; drsbadrinathan@gmail.com; 3 Center for Advanced Science & Technology and Innovation & Development Center of Sustainable Agriculture, National Chung Hsing University, Taichung 402204, Taiwan; jiannyeu.chen@nchu.edu.tw; 4 Department of Chinese Medicine, Taichung Tzu Chi Hospital, Buddhist Tzu Chi Medical Foundation, Taichung 42743, Taiwan; weec680116@gmail.com (J.-Y.L.); dr.suyu@tzuchi.com.tw (S.-Y.Y.)

**Keywords:** Kaempferitrin, *Cinnamomum osmophloeum*, proteomics, mass spectrometry, HepG2, exosome, atomic force microscopy (AFM)

## Abstract

Kaempferitrin is extracted in significantly high quantities from the leaves of *Cinnamomum osmophloeum*, which belongs to a group of plant species that comes under the genus Cinnamomum, well-known for its established anti-diabetic property in Chinese medicine. Oral administration of kaempferitrin and *Cinnamomum osmophloeum* extract reduced blood sugar in alloxan-induced diabetic rats and improved the lipid profile in hamsters respectively. In this paper we studied the differential protein expression profile using mass spectrometry approach in the kaempferitrin-treated conditioned medium of liver cancer cell line HepG2. We discovered that 33 genes were up/down-regulated consistently between two biological samples. A slightly different version of the analysis software selected 28 genes, and the final 18 genes that appeared in both lists were selected. Interestingly, 5 proteins out of 18 were either exosomal markers or reported in high frequency of occurrence in exosome/secreted vesicles. We also examined the extracellular particles with atomic force microscopy (AFM), which showed that the conditioned medium of kaempferitrin treated had larger vesicles and fewer small vesicles. Expression of some lipid-regulating genes were also altered. Our data suggested that extracellular vesicle secretions may be regulated by kaempferitrin, and regulation of lipid profile by kampeferitrin involves multiple mechanisms.

## 1. Introduction

*Cinnamomum osmophloeum* (CO) is an important plant species in traditional Chinese medicine and group of plant that comes under the same genus Cinnamomum is well-known for its anti-diabetic property. Among various bioactive compounds in CO, Kaempferitrin is a potent flavonoid compound that is extracted in significantly high quantities from the leaves [1,2,3]. Kaempferitrin is also extracted from many medicinal plants such as *Bauhinia forficata*, which are used as an antidiabetic herbal remedy in Brazil [4]. Oral administration of Kaempferitrin reduces blood sugar in alloxan-induced diabetic rats [4,5], and intraperitoneal injection of polyphenols extracted from *Cinnamomum osmophloeum* leaves reduces blood sugar in diabetic rats [6].

Commercial product using dry *Cinnamomum osmophloeum* leaves have been sold locally in Taiwan. Though previously demonstrated to activate the classical insulin signaling pathways, mechanism for anti-diabetic activity of kaempferitrin is still not clearly understood. Furthermore, as references for kaempferitrin on immune cells and related mechanisms have been inconclusive, and people consuming extract containing kaempferitrin often happen to be at high risk of diabetes and neurodegenerative diseases, comprehensive evidence are needed in support of daily supplementation of kaempferitrin for any disorder. We have recently published a paper in which we used the proteomic approach to study the differential protein expression after kaempferitrin treatment in astrocytic cells [7]. By utilizing dimethyl labelling on the peptide level and LC-MS/MS, we were able to quantitate 13,013 peptides, corresponding to 1264 proteins from two biological replicates. Total of 32 differentially expressed proteins were shortlisted, which include LDL-R and IGFBP2.

After confirming that the immune response was not provoked by kaempferitrin, we found several genes that were interesting among the regulated proteins. A group of genes involved in apoptosis were significantly regulated in the secretome. Other regulated proteins include LDL-R and IGFBP2, both very relevant to the metabolic homeostasis, were down-regulated in the astrocytic secretome. Their expression was confirmed by Western blots [7]. After careful search in the literature, we found that presence of proteins from intracellular origin in the secretome are not due to cell death or serum contamination, as it has been demonstrated that intracellular proteins possess alternative functions in the extracellular compartment and hence, could be transported to the outside of the cells via unconventional transport (bypass Golgi for example) or by exosome [8]. Hepatocytes are primarily responsible for the regulation of lipid generation systemically. It is the center of fatty acid synthesis and lipid circulation from/to extra hepatic tissues through lipoprotein metabolism [9]. *Cinnamomum osmophloeum* leaf extracts was known to contain kaempferitrin, and oral administration of CO leaf extracts to hyperlipidemic hamsters reduced their total cholesterol, triglyceride, and low-density lipoprotein (LDL-C) levels [10]. This leads to our speculation that kaempferitrin might play a certain role in hepatocytes in regulating protein expression in the intracellular compartment and secretome, relevant to metabolism in other insulin-sensitive or lipid-metabolizing cells. Hence, we intended to study the effect of kaempferitrin on HepG2 cells and molecular profile of its extracellular vesicles (EVs) content that take part in modulation of lipid metabolism and related genes. HepG2 cells were chosen for the experiments because of its relevance in endogenous lipid generation and we reported an interesting proteomic issue for hepatocyte-secreted vesicle, through two different approaches (Western blots and AFM), to explain the results. Our observation may provide a better understanding in diagnosis and management of several liver complications mediated by obesity, fatty liver disease, and steatohepatitis culminating in hepatocellular carcinoma and other extrahepatic abnormalities.

## 2. Materials and Methods

### 2.1. Cell Culture, Drug Treatment, and Sample Collection for Proteomics and Western Blotting

HepG2 cells were cultured in DMEM supplemented with 4 mM glutamine and 10% fetal bovine serum without the addition of phenol red for 24 h. The cells were rinsed thoroughly with PBS to remove any traces of serum, followed by incubation in DMEM + 4 mM glutamine (no phenol red) for 24 h. The cells in the test group were treated with 10 μM kaempferitrin in the same medium for another 24 h, and cells that were not treated with kaempferitrin were used as a control group. At the end of 24 h, the conditioned medium was collected, passed through a 0.22-μm filter to remove cell debris, and concentrated in an Amicon column with a 3-KD cutoff (Millipore, Billerica, MA, USA) via centrifugation at 11,000× *g*. The concentration step was accompanied by several rounds of washing with PBS to facilitate desalting. The eluate was frozen before proteomics study. For Western blot, the cells were cultured by method similar to the proteomic study, but after the conditioned medium was collected, some of them were passed through 0.22 μM filter and some through 0.45 μm (discussed in detail in the following sections).

### 2.2. Quantitative Proteomics Using Dimethyl Labelling

Secreted proteins were first re-suspended in a digestion buffer (100 mm triethylammonium bicarbonate and 8 M urea), S-alkylated, and trypsin digested as previously described [7]. Dimethyl peptide labelling was performed according to the method established by Boersema et al. [11]. In brief, trypsin-digested peptides from kaempferitrin-treated and control-conditioned media were labelled with stable-isotopic formaldehyde (^13^CD_2_O, heavy-labelled) and formaldehyde (CH_2_O, light-labelled), respectively. Equal amounts of the heavy- and light-labelled peptides were mixed, desalted, and subjected to StageTip-based strong cation-exchange fractionation [12] with sequential elution by increasing salt concentrations (30, 60, 90, 200, and 500 mM ammonium acetate in 15% acetonitrile and 0.1% trifluoroacetic acid).

The eluted peptide fractions were desalted and analyzed by nanoflow liquid chromatography-tandem mass spectrometry (nanoLC-MS/MS) as previously described by Ku et al. [7] The peptides were loaded and resolved by Dionex Ultimate 3000 RSLC nano system (Thermo Fisher Scientific, Bremen, Germany) with in-house-prepared 100 μm × 15 cm tip column packing with 3-μm ReproSil-Pur 120 C18-AQ reverse-phase beads (Dr. Maisch HPLC GmbH, Ammerbuch-Entringen, Germany). The eluted peptides were then analyzed by online-coupled LTQ Orbitrap XL mass spectrometer (Thermo Fisher Scientific) operating in data-dependent acquisition mode. In our study, we analyzed two independent biological batches in triplicate LC-MS/MS runs for each SCX fraction.

### 2.3. Data Analyses

Protein identification and quantitation were performed using MaxQuant software (version 1.6.14.0) [13] against SWISS-PROT sequence database (version 2019_10 with 20,442 canonical human protein sequence entries) with peptide identification setting as previously described by Ku et al. [7]. False discovery rates (FDRs) at the peptide and protein levels were fixed at 1%. Quantitative information (treatment/control or H/L) corresponding to each protein was calculated and log2 transformed with at least two quantifiable peptides using MaxQuant. The potential up- or down-regulated protein candidates were filtered with Perseus software (version 1.6.0.7) by applying student’s cut-off thresholds *p* < 0.05 followed by further filtering for log2 ratio >0.5 or <−0.5. All LC-MS/MS raw data and MaxQuant search result were deposited to the ProteomeXchange Consortium via the PRIDE partner repository with the dataset identifier PXD022181.

The protein candidates that were similarly up-regulated or down-regulated in the two biological batches were selected (shown in Table 1). The data were analyzed again using a latest version of MaxQuant software (shown in Table 2). Out of the candidate’s proteins displayed in Table 1 and Table 2, those that were selected by both analyses was kept in the final list (shown in Table 3).

### 2.4. Atomic Force Microscopy (AFM)

To test whether the size and number of EVs are altered upon kaempferitrin treatment, we performed atomic force microscopy (AFM) (Nanoview1000, FSM–Precision) of the control- and kaempferitrin-conditioned medium after filtration. Tapping mode was used, and 3000 nm × 3000 nm area in channels including phase and amplitude were recorded. For analyzing extracellular vesicles number and particle size, images were obtained from two points on the mica. Two images were taken from each mica for three experiments of different dates, and size of the vesicles was measured using built-in software (Nanoview1000, FSM–Precision) with enlarged images. Some samples were photographed using another AFM (Bruker Dimension Icon). The HepG2 cells were cultured in a similar way to those for proteomics, except 0.45 μm filter was used in an attempt to retain EVs in the medium. They were frozen and stored until use. After defrost, the collected conditioned medium was condensed and desalted by 3D flow through a filter (Amicon). They were diluted 100 times with double distilled water, and 10 uL of the diluted medium was spotted on the mica and allowed to air dry.

## 3. Results

### 3.1. Proteomics

#### 3.1.1. First Round Selection

In this study, we used HepG2 as a model to analyze the differentially regulated secretomes by kaempferitrin. By utilizing dimethyl labeling on the peptide level and LC-MS/MS, we were able to quantitate 13,013 peptides, which correspond to 1264 proteins from two independent biological replicates. The protein expression ratio (Kaempferitrin vs. control) was first averaged, and log2 transformed. The potential kaempferitrin-regulated proteins were filtered based on student’s t-test with the threshold value *p* < 0.05, and further filtered for log2 ratio >0.5 and <−0.5 for up- and down-regulated proteins, respectively. The initial filtering allowed us to find 67 kaempferitrin-regulated proteins. In order to increase the data confidence, only the regulated proteins showing the same trends in two biological replicates were selected. The resulting 33 regulated proteins were selected (Table 1).

#### 3.1.2. Second Round Selection

The same set of data was analyzed again using a later version of the same software, as well as using an updated protein database (shown in Table 2). With this algorithm. The initial filtering and subsequent selection of regulated proteins showed the same trends in two biological replicates. A list of 28 candidates was obtained (Table 2).

#### 3.1.3. Double selection

Out of the candidates that were selected from Table 1 and Table 2, those that were selected by both analyses were kept in the final list of 18 proteins (shown in Table 3).

### 3.2. Identification of Exosomal Markers

We compared our list of 18 shortlisted proteins to the top 100 exosomal marker compiled by Dr. Suresh Mathivanan http://exocarta.org/exosome_markers_new and found that 5 were expressed in EVs with high frequency (Table 4). This includes (data presented in gene names, followed by protein name, and the number of times identified versus highest score presented for exosomal marker in parenthesis): HSP90AA1 (heat shock protein HSP 90-alpha, 77/98), YWHAZ (14-3-3 protein zeta/delta) (69/98), HSP90AB1 (heat shock protein HSP 90-beta, 67/98), YWHAE (14-3-3 protein epsilon) (65/98), UBA1 (ubiquitin-like modifier-activating enzyme) (45/98). These exosomal markers were all consistently down-regulated. The fold change of protein expression level was very consistent around 0.67 for all the 5 exosomal markers.

### 3.3. Western Blots

#### 3.3.1. The 0.22-μm Filter

**Figure 1:**
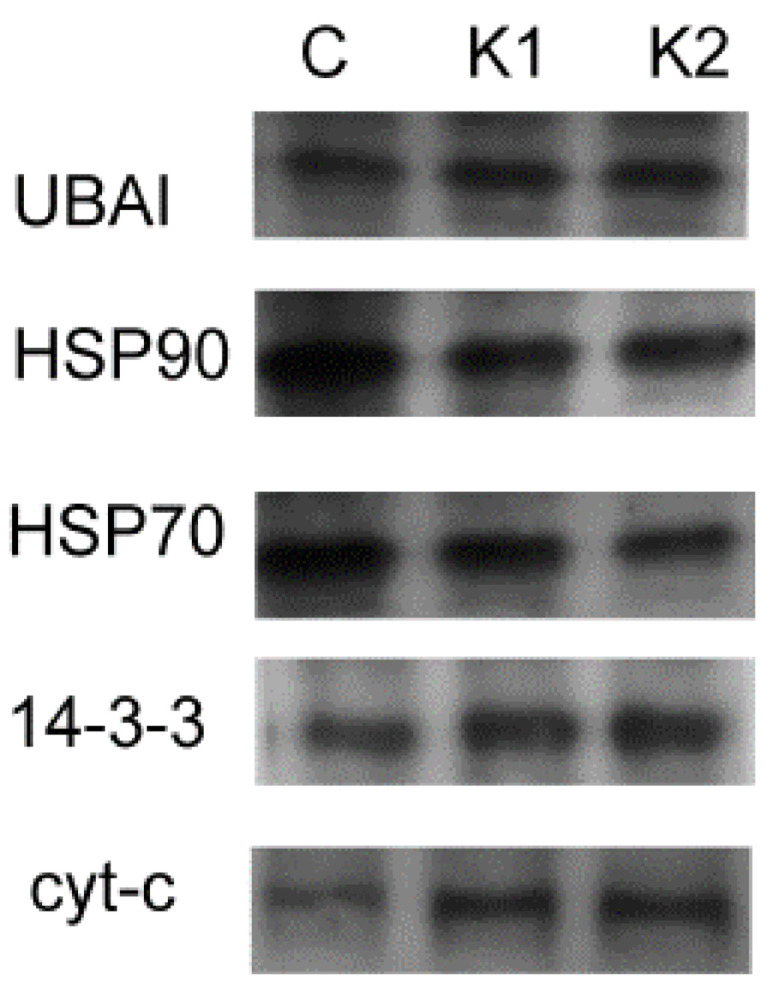
Western blots for the five exosomal markers that were demonstrated as differentially regulated by the proteomic methods. The conditioned medium was filtered with a 0.22-μm membrane.

To verify the results from the proteomics, we treated the HepG2 cells with 10 μm kaempferitrin, collected and filtered the conditioned medium, 0.22 μm and concentrated with 3D flow through column. Among the five differentially expressed exosomal marker proteins, we tested the UBA1, HSP90a, and 14-3-3 epsilon. We also tested HSP70, as the known marker for the exosome for its expression and Cytochrome C protein levels. According to our observation from Figure 1, HSP90a and HSP70 were down-regulated, which corroborated with the proteomic data, while UBA1 was up-regulated in both samples, and 14-3-3-epsilon was up-regulated in one of the samples, which did not corroborate with the proteomic data. Cytochrome C was up-regulated as reported in the proteomic analysis.

#### 3.3.2. The 0.45-μm Filter

**Figure 2:**
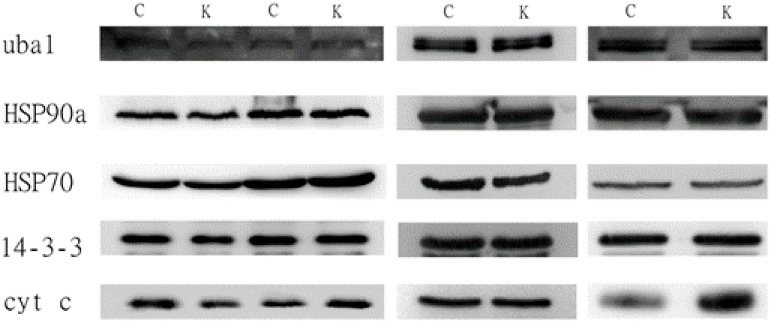
Western blots for the five exosomal markers that were demonstrated as differentially regulated by the proteomic methods. The conditioned medium was filtered with 0.45-µm membrane.

We prepared the conditioned medium that was filtered with a 0.45-μm membrane instead of the 0.22 µm to be tested with Western blots. However, down-regulation pattern of HSP90a and HSP70 was not observed (Table 5) and the exosomal marker levels were very similar between the treated and the untreated samples (Table 5 and Figure 2).

### 3.4. AFM Studies

#### 3.4.1. Nanoview1000

**Figure 3:**
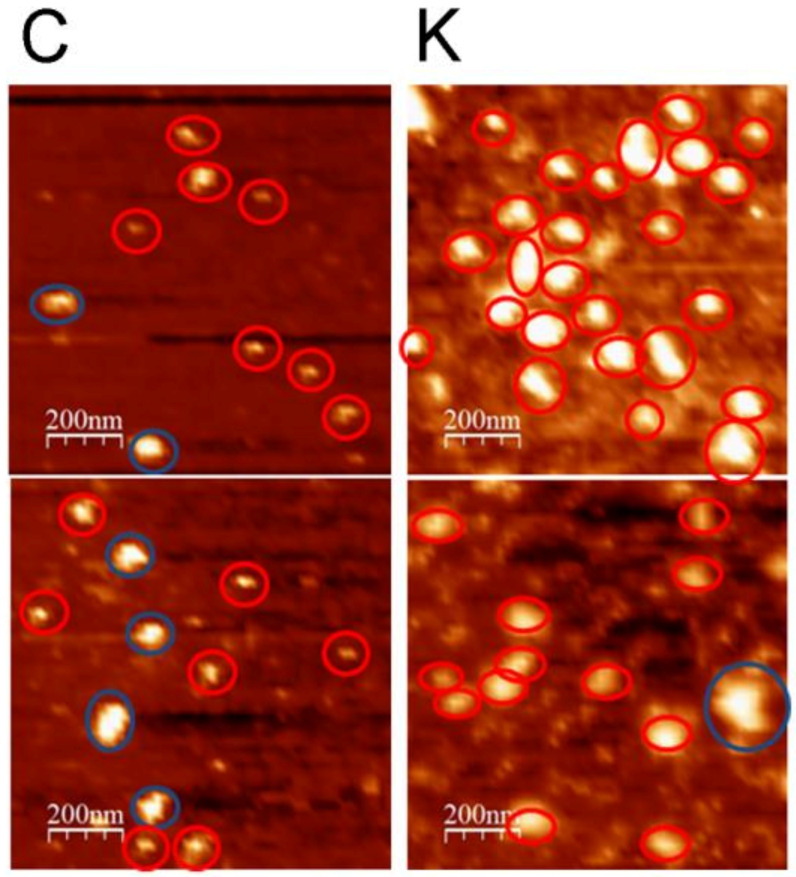
Representative photos for extracellular vesicles (EVs) viewed under Nanoview 1000. Column C: controls. Column K: kaempferitrin treated. Two views were presented for each treatment.

A representative photograph of the view from the Nanoview 1000 was provided in Figure 3. The data from the scan in the Nanoview 1000 were pooled from three experiments and divided according to their size into two groups: >100 nM, and <100 nM. The number and size of the EVs were analyzed and listed in the following Table 6 and Table 7. The data indicated that, among all the EVs examined in the pooled data from three experiments and two images each, kaempferitrin-treated samples have more particles, and the sizes were larger when treated with kaempferitrin. The *p*-value from the t test was 0.076 which was not significant, but it showed an increasing tendency. When the particles that were smaller than 100 nm in diameter were analyzed, the control sample showed much smaller particles than the kaempferitrin-treated, which could be significantly tested different by student t test and this was supported by average size of EVs, which was smaller in the control than the kaempferitrin-treated medium.

#### 3.4.2. Brucker Dimension Icon

**Figure 4:**
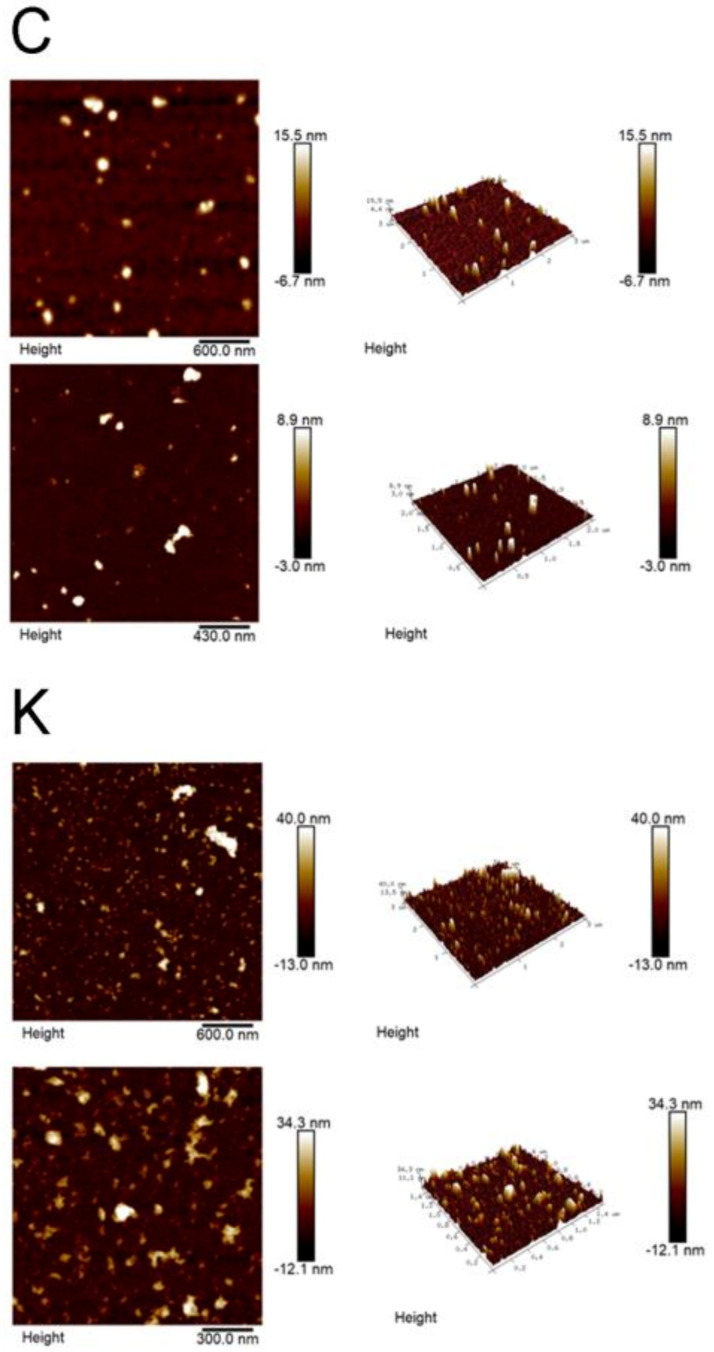
Representative photos for EVs viewed under Brucker Dimension Icon. C: Controls. K: kaempferitrin treated. Two views were presented for each treatment. The left columns are the height channels. The middle column shows the height in the 3D configuration with slight tilt in the *xy*-axis. The white to black scales demonstrate the measurement of height in *Z*-axis and was represented with the white designated for the highest point. Note the height of the EVs on the *Z* axis was 15.5 and 8.9 nm while those for the kaempferitrin-treated are 40.0 and 34.3 nm.

Some samples were taken from the conditioned medium collected and filtered with 0.45 um for Western blots and scanned using another AFM machine (Brucker Dimension Icon) (Figure 4). The resolution in this machine is higher than Nanoview 1000, and it also provides the *Z* axis information. The height on the *Z* axis for the control was 15.5 and 8.9 nm, respectively, for the area we chose to analyze. The height on the *Z* axis for the kaempferitrin-treated were 40 and 34.3 nm, which were significantly higher than the control. Although size of the exosome was normally about 30–150 nm, it does not imply any error in our measurement. Our preparation involved air drying the sample on mica and therefore could provide extra pressure to the exosome particle when the liquid evaporating around the exosome.

As a consolidated observation, our data revealed that the kaempferitrin treatment results in larger exosomal particles. It also resulted in a smaller number of the smaller particles (<100 nm).

## 4. Discussion

### 4.1. Regulation of Exosomal Markers

Significantly high percentage of the proteins that were regulated among the final 18 candidates were exosomal markers. Five out of the 18 corresponds to roughly 28% and based on this information on peptide frequency that were identified (Table 1, peptides), the absolute quantity is much higher. These altered exosomal proteins that were significantly expressed in high frequency, surprisingly showed a similar degree of down-regulation to 0.67-fold of the control sample. This suggests that the difference in protein species and quantity we detected between treatments was partially due to the difference in EVs’ population. Furthermore, this is not a general down-regulation of total proteins, as the cytochrome C proteins, which is a marker for mitochondria or apoptosis was up-regulated.

### 4.2. The Kaempferitrin Treatment Results in Alteration to the EVs

If we divide the vesicle size into two groups, >100 nm or <100 nm, the kaempferitrin resulted in larger size in larger particle populations (>100 nM) and larger size in smaller particle populations (<100 nm). There are significantly less exosmal particles in the <100 nm population (less than half of control). In other words, they were increased in diameter for both sizes, and there are fewer smaller particles. The 0.22-µm filter we used, in our experience, did not filter so precisely that anything smaller than 220 nmM will flow through. The cut-off size may vary because of the g force we applied, as well the time of centrifugation, or whether the exosomes were aggregated, which was reported by other studies. It was reported that aggregates were quite common and could be solved by the addition of detergent like tween-20, which we do not have in our suspension. Therefore, we consider that larger size may raise the chance of being retained during filtration, and hence the difference in the protein ratio we tested in the proteomics.

### 4.3. Western Blot Result for Conditioned Medium

From the two Western blot samples that were filtered through 0.22-µm (Figure 1), we saw that the HSP90a and the cytochrome C results supported the proteomic data while UBA1 and 14-3-3 epsilon, on the other hand, did not corroborate. We have tested HSP70, the most popular exosomal marker, and it was down-regulated in the treated medium. When the conditioned medium was filtered with a larger 0.45-µm membrane, the difference in the protein levels between the treated and the non-treated conditioned medium was not observed. At the same time, the same conditioned medium, when observed down the AFM, demonstrated a larger-sized EV and significantly reduced number of small EVs. This has led us to speculate that the difference in protein levels for the exosomal proteins we saw in the proteomic data was actually a result of the 0.22-µm filter devoid of the larger EVs from the conditioned medium.

### 4.4. Why Are Kaempferitrin Treated EVs Larger?

The endosomal sorting complexes required for transport (ESCRT) machinery is made up of cytosolic protein complexes. Together with a number of accessory proteins, these ESCRT complexes enable a unique mode of membrane remodeling that results in membranes bending/budding away from the cytoplasm. VPS4A is one of the components of ESCRT complex and it inhibits the production of HCC-related exosome by acting on cell-bound ATP to provide energy [14]. This information suggests that enzyme that act on cell-bound ATP to provide energy play a role during membrane budding from the cytoplasm. Interestingly, kaempferol, a molecule with structure similar to kaempferitrin, was known to boost the membrane-bound ATPase activity [15]. As previously discussed in our publication [16], kaempferitrin does not directly bind to insulin receptor but was able to influence its phosphorylation and down-stream signaling. Therefore, its action must be around the vicinity of the cell membrane. It would be interesting to see in the future if the kaempferitrin regulates ATPase activities like kaempferol does, and affect the membrane assembly for exosomes, which might be the mechanism regulating the exosome size.

### 4.5. Regulation of Lipid Metabolism

We observed the up-regulation of Apolipoprotein C-II/Pro-apolipoprotein C-II and down-regulation of bile salt sulfotransferase in the conditioned medium upon kaempferitrin treatment. Apolipoprotein C is a component of VLDL, HDL, and chylomicrons, and known to activate extrahepatic lipoprotein lipase (LPL), as well as lipoproteins located in endothelial cells of small blood vessel and capillaries [17]. ApoC-II plays a critical role in triglyceride-rich lipoproteins (TRL) metabolism by acting as a cofactor of lipoprotein lipase (LPL), the main enzyme that hydrolyses plasma triglycerides (TG) in TRL. After the action of the LPL, these lipids were turned into chylomicron remnants, which enters the lymphatic system, and endocytosed to enter liver. LPL was therefore called “clearing factor” [18]. Bile salt sulfotransferase is also known as hydroxysteroid sulfotransferase (HST) or sulfotransferase 2A1. Sulfotransferase utilizes 3′-phospho-5′-adenylyl sulfate (PAPS) as sulfonate donor to catalyze the sulfonation of steroids and bile acids in the liver and adrenal glands. These were the two proteins that are specifically activated in liver cells [19], as we did not observe a regulation of these two proteins when astrocytic cell lines were treated with kaempferitrin. These might be relevant in explaining the kaempferitrin effect on the lipid metabolism in mice fed with kaempferitrin.

As previously described, many metabolic relevant proteins were regulated by kaempferitrin, and it was less likely that one chemical, with its finite structure, could interact with so many molecules, unless it controls one single master gene, or otherwise, one cellular process that involved general regulation of proteins. In this case the post-translational unconventional transport or exosome release, might provide an interesting hypothesis. In fact, kaempferitrin has shown to increase the glucose uptake in rat soleus muscle and reduce the blood glucose level [4,20,21], and to inhibit the lipid peroxidation and clear oxidative stress [2,4,22], and possess insulin mimetic ability [16,22]. Oral administration of *Cinnamomum osmophloeum* leaf extracts, which contain significant quantity of kaempferitrin, improved the hyperlipidemic condition in higher animal models like hamsters, by reducing their total cholesterol, triglyceride, and low-density lipoprotein (LDL-C) levels [10].

### 4.6. Proteomic as a Tool for Study Exososomal Regulation

Mass spectrometry as a tool for proteomic analysis has proved to be a powerful and convincing methodology. It produces data that report differences in large quantity of protein species. However, sometimes the sheer quantity could be problematic if one is not familiar with the biological system and did not understand and interpret the data wisely. Secretome, unlike the cytoplasmic content, contains less protein species and may not be very challenging a system. The study of exosomes, or more accurately put, excreted vesicles, attract great interest as it can be used as a drug-delivery system, as well as a good biomarker for cancer and other chronic diseases. However, exosomal study suffers from a lack of definite markers and the vesicle should possess at least three different marker protein to be identified as exosomes, and still there are some secreted EVs that did not express the most common markers. Therefore, the use of proteomic tools to provide a profile of exosomal marker may benefit the exosomal study in which the marker profiling might be a challenging task.

## 5. Conclusions

The results from our paper suggested that it is useful to pick up differences in exosomal markers. The ability to retain details benefit the study of EVs, as this group of vesicles varies in its content and marker proteins. As more than few markers could be consistently used to define these vesicles, an array of protein information is advantageous, therefore differential mass spectrometry may be a good tool for exosomal studies. This research presents an interesting proteomic profile for hepatocyte-secreted vesicle, and we tried to explain it using two different approaches. Kaempferitrin is much well-known for its insulin mimetic effect, and it is already known that it reduces blood lipid in rats, but the mechanism was not known. Observations from this paper could possibly lead to further understanding of its lipid regulating mechanism that plays a pivotal role in the progression of several hepatic and extrahepatic diseases mediated through extracellular vesicles.

## Figures and Tables

**Table 1:** First round of protein selection.

Majority Protein IDs	Protein Names	Gene Names	Peptides
Q8NCF0; A5D8T8; Q6UXF7	C-type lectin domain family 18 member C; C-type lectin domain family 18 member A; C-type lectin domain family 18 member B	*CLEC18C*; *CLEC18A*; *CLEC18B*	1
A6NC57	Ankyrin repeat domain-containing protein 62	*ANKRD62*	1
O95433	Activator of 90 kDa heat shock protein ATPase homolog 1	*AHSA1*	3
P02655	Apolipoprotein C-II; Proapolipoprotein C-II	*APOC2*	2
P06748	Nucleophosmin	*NPM1*	8
P07900	Heat shock protein HSP 90-α	*HSP90AA1*	27
P08238	Heat shock protein HSP 90-β	*HSP90AB1*	29
P08243	Asparagine synthetase [glutamine-hydrolyzing]	*ASNS*	1
P0DP25; P0DP24; P0DP23	Calmodulin-3, Calmodulin-2, Calmodulin-1	*CALM3*, *CALM2*, *CALM1*	7
P13473	Lysosome-associated membrane glycoprotein 2	*LAMP2*	1
P14174	Macrophage migration inhibitory factor	*MIF*	1
P14868	Aspartate-tRNA ligase, cytoplasmic	*DARS*	5
P16422	Epithelial cell adhesion molecule	*EPCAM*	2
P22314	Ubiquitin-like modifier-activating enzyme 1	*UBA1*	12
P28799	Granulins; Acrogranin; Paragranulin; Granulin-1; Granulin-2; Granulin-3; Granulin-4; Granulin-5; Granulin-6; Granulin-7	*GRN*	8
P31946	14-3-3 protein β/α;14-3-3 protein β/α, N-terminally processed	*YWHAB*	6
P39060	Collagen α-1(XVIII) chain; Endostatin	*COL18A1*	1
P50897	Palmitoyl-protein thioesterase 1	*PPT1*	5
P51857	3-oxo-5-β-steroid 4-dehydrogenase	*AKR1D1*	1
P62258	14-3-3 protein epsilon	*YWHAE*	12
P63104	14-3-3 protein zeta/delta	*YWHAZ*	7
P68402	Platelet-activating factor acetylhydrolase IB subunit β	*PAFAH1B2*	1
P99999	Cytochrome c	*CYCS*	4
Q06520	Bile salt sulfotransferase	*SULT2A1*	3
Q07955	Serine/arginine-rich splicing factor 1	*SRSF1*	3
Q16576; Q09028	Histone-binding protein RBBP7; Histone-binding protein RBBP4	*RBBP7*; *RBBP4*	2
Q58FF8	Putative heat shock protein HSP 90-β 2	*HSP90AB2P*	6
Q8TDY2	RB1-inducible coiled-coil protein 1	*RB1CC1*	1
Q92688	Acidic leucine-rich nuclear phosphoprotein 32 family member B	*ANP32B*	3
Q92896	Golgi apparatus protein 1	*GLG1*	1
Q969E1	Liver-expressed antimicrobial peptide 2	*LEAP2*	1
Q99832	T-complex protein 1 subunit eta	*CCT7*	3

**Table 2:** Second round of selection.

Majority Protein IDs	Protein Names	Gene Names	Peptides
A6NC57	Ankyrin repeat domain-containing protein 62	*ANKRD62*	1
P02655	Apolipoprotein C-II; Proapolipoprotein C-II	*APOC2*	2
P07900	Heat shock protein HSP 90-α	*HSP90AA1*	27
P08238	Heat shock protein HSP 90-β	*HSP90AB1*	29
P08833	Insulin-like growth factor-binding protein 1	*IGFBP1*	12
P0DP25; P0DP24; P0DP23			7
P14174	Macrophage migration inhibitory factor	*MIF*	1
P22314	Ubiquitin-like modifier-activating enzyme 1	*UBA1*	12
P26641	Elongation factor 1-γ	*EEF1G*	6
P28799	Granulins; Acrogranin; Paragranulin; Granulin-1; Granulin-2; Granulin-3; Granulin-4; Granulin-5; Granulin-6; Granulin-7	*GRN*	8
P39060	Collagen α-1(XVIII) chain; Endostatin	*COL18A1*	1
P51857	3-oxo-5-β-steroid 4-dehydrogenase	*AKR1D1*	1
P62258	14-3-3 protein epsilon	*YWHAE*	12
P63104	14-3-3 protein zeta/delta	*YWHAZ*	7
P68402	Platelet-activating factor acetylhydrolase IB subunit β	*PAFAH1B2*	1
P84103	Serine/arginine-rich splicing factor 3	*SRSF3*	2
P99999	Cytochrome c	*CYCS*	4
Q06033	Inter-α-trypsin inhibitor heavy chain H3	*ITIH3*	7
Q06520	Bile salt sulfotransferase	*SULT2A1*	3
Q07955	Serine/arginine-rich splicing factor 1	*SRSF1*	3
Q16576; Q09028	Histone-binding protein RBBP7; Histone-binding protein RBBP4	*RBBP7*; *RBBP4*	2
Q58FF8	Putative heat shock protein HSP 90-β 2	*HSP90AB2P*	6
Q5R3I4	Tetratricopeptide repeat protein 38	*TTC38*	3
Q6UW63	KDEL motif-containing protein 1	*KDELC1*	3
Q92563	Testican-2	*SPOCK2*	9
Q92688	Acidic leucine-rich nuclear phosphoprotein 32 family member B	*ANP32B*	3
Q96FW1	Ubiquitin thioesterase OTUB1	*OTUB1*	3
Q9Y4L1	Hypoxia up-regulated protein 1	*HYOU1*	4

**Table 3:** Proteins that were selected by both versions.

Majority Protein IDs	Protein Names	Gene Names	Peptides
A6NC57	Ankyrin repeat domain-containing protein 62	*ANKRD62*	1
P02655	Apolipoprotein C-II; Proapolipoprotein C-II	*APOC2*	2
P07900	Heat shock protein HSP 90-α	*HSP90AA1*	27
P08238	Heat shock protein HSP 90-β	*HSP90AB1*	29
P14174	Macrophage migration inhibitory factor	*MIF*	1
P22314	Ubiquitin-like modifier-activating enzyme 1	*UBA1*	12
P28799	Granulins; Acrogranin; Paragranulin; Granulin-1; Granulin-2; Granulin-3; Granulin-4; Granulin-5; Granulin-6; Granulin-7	*GRN*	8
P39060	Collagen α-1(XVIII) chain; Endostatin	*COL18A1*	1
P51857	3-oxo-5-β-steroid 4-dehydrogenase	*AKR1D1*	1
P62258	14-3-3 protein epsilon	*YWHAE*	12
P63104	14-3-3 protein zeta/delta	*YWHAZ*	7
P68402	Platelet-activating factor acetylhydrolase IB subunit β	*PAFAH1B2*	1
P99999	Cytochrome c	*CYCS*	4
Q06520	Bile salt sulfotransferase	*SULT2A1*	3
Q07955	Serine/arginine-rich splicing factor 1	*SRSF1*	3
Q16576; Q09028	Histone-binding protein RBBP7; Histone-binding protein RBBP4	*RBBP7*; *RBBP4*	2
Q58FF8	Putative heat shock protein HSP 90-β 2	*HSP90AB2P*	6
Q92688	Acidic leucine-rich nuclear phosphoprotein 32 family member B	*ANP32B*	3

**Table 4:** Exosomal markers that were selected in the final list.

Majority Protein IDs	Protein Names	Gene Names
P07900	Heat shock protein HSP 90-α	*HSP90AA1*
P08238	Heat shock protein HSP 90-β	*HSP90AB1*
P22314	Ubiquitin-like modifier-activating enzyme 1	*UBA1*
P62258	14-3-3 protein epsilon	*YWHAE*
P63104	14-3-3 protein zeta/delta	*YWHAZ*

**Table 5:** The ratio of protein levels of kaempferitrin treated sample over non-treated samples tested by Western blots for conditioned medium prepared with 0.22-µm or 0.45-µm filter.

K/C Ratio	Filter (µm)	UBA1	HSP90A	HSP70	14-3-3 Epsilon	Cytochrome c
sample 1	0.22	1.27	0.71	0.50	1.04	1.25
sample 2	0.22	1.39	0.69	0.35	0.83	1.37
sample 3	0.45	0.96	0.87	0.87	0.99	1.10
sample 4	0.45	0.85	0.94	0.99	1.00	1.77
sample 5	0.45	0.93	1.17	0.90	0.98	0.86
sample 6	0.45	0.91	0.99	0.92	0.99	1.24

**Table 6:** Particles that have diameter >100 nm.

	Control	K ^1^	t Test
EVs number	33	49	0.076379451
Average size (nm)	131.84	146.02	

^1^ K: kaempferitrin treated.

**Table 7:** Particle size that is <100 nm.

	Control	K ^1^	t Test
EVs number	101	39	0.00005
Average size (nm)	64.72	77.44	

^1^ K: kaempferitrin treated.

## Data Availability

All LC-MS/MS raw data and MaxQuant search result were deposited to the ProteomeXchange Consortium via the PRIDE partner repository with the dataset identifier PXD022181.
